# The Illinois Board of Health

**Published:** 1887-07

**Authors:** 


### THE ILLINOIS BOARD OF HEALTH.

THE regular quarterly meeting of the Illinois State Board of Health was held in Chicago on the 8th instant. There were present Drs. W. A. Haskell, Alton, President ; A. L. Clark, Elgin ; G. N. Kreider, Springfield ; W. H. Mackenzie, Chester ; and Dr. John H. Rauch, Chicago, Secretary. The Secretary called attention to the terms of the act passed by the last General Assembly, and moved that the members proceed to organize in accordance therewith, the officers and committees of the old organization to be continued. The motion was carried.

The following resolution was adopted:

"*Resolved*, That this board reaffirms the action of the State Board of Health taken May 2, 1885, in revoking the certificate of John C. McCoy on charges of unprofessional and dishonorable conduct then presented and the findings against him on such charges."

The Secretary presented the applications of sundry itinerant venders of drugs, nostrums, etc., eight in number. After a full discussion of the subject Dr. Kreider offered the following resolution, which was adopted:

"*Resolved*, That the applications now pending by itinerant venders of drugs, nostrums, etc., for license to sell and the tender of the fee of $100 per month for each such license be declined in the exercise of the discretion vested in this board by Sec. 11 of the Act to regulate the Practice of Medicine, such itineracy and the manner of vending being deemed prejudicial to the public welfare."

Dr. Mackenzie offered the following, which was also adopted :

"*Resolved*, That conviction of the charge of criminal abortion performed or procured by a practitioner of medicine or midwife shall be held as cause for the refusal or summary revocation of the certificate of this board."

Dr. John A. Rauch, Secretary of the board, reported that there were issued to graduates upon diplomas from medical colleges in good standing 154 certificates since the last meeting of the Board, and to six others on examination ; also four to non-graduates upon proof of twenty-one years' practice in the state, and one duplicate. Fifteen applications for certificates were rejected through inability of applicants to comply with the requirements. Certificates were issued to twenty-eight midwives. Few laws have done more for the practical welfare of the community than the Act to Regulate the Practice of Medicine in Illinois for the last ten years, and the brief record was the best earnest for the future of the faithful and conscientious discharge of the added responsibilities devolved upon the board by the new act approved June 17, 1887. The original law took effect July 1, 1877, and by its terms the following six months were allowed in which to comply with its requirements. During this period it was learned that there were in round numbers July 1, 1877, an aggregate of 7,400 persons engaged in the practice of medicine in Illinois. Of these about 3,800, or more than half, were non-graduates, and these comprised all classes, including those who had assumed the title of "Doctor" without any medical study whatever. The following table gives a comparative exhibit of the status of the profession at the beginning and at the end of these ten years. Comparison of the total number and classes of physicians in the state July 1, 1877, and July 1, 1887:

| | July 1, 1877. | July 1, 1887. |
|---|---|---|
| Total number engaged in practice | 7,400 | 6,180 |
| Graduates and licentiates | 3,600 | 5,704 |
| Non-graduated | 3,800 | 476 |
| Percentage of graduates and licentiates | 48.6 | 92.3 |
| Percentage of non-graduates | 51.4 | 7.7 |

When the law went into effect there were 1,923 physicians in the state who were not qualified and therefore could not comply with its provisions. Of these by far the greatest number left the state, others abandoned practice, while many qualified themselves and graduated or passed the examinations of the board. In addition the board refused during the year 1878 278 applications for certificates ; in 1879, 175; 1880,154; 1881,127; 1882,111; 1883,98; 1884, 185; 1885, 75; 1886,73; and thus far in 1887, 25, making a total of 3,129. Since the organization about 2,000 complaints involving the professional conduct of the licentiates of the board have been received. Nearly all were settled. Thus far this season the conditions affecting the public heath have been quite satisfactory, there being nothing in the state to excite public uneasiness. Occasional small-pox cases have occurred, and measles has been the prevailing sickness in many sections. Yellow-fever at Key West has thus far been confined to that quarter, and carefulness here will better fortify us against any danger from cholera.

Dr. Clarke offered the following resolution, which was adopted:

"*Resolved*, That the phrase "medical colleges in good standing" in the first section of the act to regulate the practice of medicine, approved June 16, 1887, is hereby defined to include only those colleges which shall, after the sessions of 1890–'91, require four years of professional study, including any time spent with a preceptor and three regular courses of lectures, as conditions of graduation, and shall otherwise conform to the schedule of minimum requirements heretofore adopted by the board."

The Secretary was instructed to prepare forms of weekly mortality reports for the use of localities where returns of death are made.

